# SILAC-based quantitative proteomics to investigate the eicosanoid associated inflammatory response in activated macrophages

**DOI:** 10.1186/s12950-022-00309-8

**Published:** 2022-09-01

**Authors:** Nicole Brace, Ian L. Megson, Adriano G. Rossi, Mary K. Doherty, Phillip D. Whitfield

**Affiliations:** 1 Division of Biomedical Sciences, University of the Highlands and Islands, Centre for Health Science, Old Perth Road, Inverness, IV2 3JH UK; 2 Centre for Inflammation Research, The Queen’s Medical Research Institute, University of Edinburgh, 47 Little France Crescent, Edinburgh, EH16 4TJ UK; 3 Present Address: Glasgow Polyomics, Garscube Campus, University of Glasgow, Glasgow, G61 1BD UK

**Keywords:** Gene ontology, Inflammation, Prostaglandins, Proteomic, RAW 264.7

## Abstract

**Background:**

Macrophages play a central role in inflammation by phagocytosing invading pathogens, apoptotic cells and debris, as well as mediating repair of tissues damaged by trauma. In order to do this, these dynamic cells generate a variety of inflammatory mediators including eicosanoids such as prostaglandins, leukotrienes and hydroxyeicosatraenoic acids (HETEs) that are formed through the cyclooxygenase, lipoxygenase and cytochrome P450 pathways. The ability to examine the effects of eicosanoid production at the protein level is therefore critical to understanding the mechanisms associated with macrophage activation.

**Results:**

This study presents a stable isotope labelling with amino acids in cell culture (SILAC) -based proteomics strategy to quantify the changes in macrophage protein abundance following inflammatory stimulation with Kdo2-lipid A and ATP, with a focus on eicosanoid metabolism and regulation. Detailed gene ontology analysis, at the protein level, revealed several key pathways with a decrease in expression in response to macrophage activation, which included a promotion of macrophage polarisation and dynamic changes to energy requirements, transcription and translation. These findings suggest that, whilst there is evidence for the induction of a pro-inflammatory response in the form of prostaglandin secretion, there is also metabolic reprogramming along with a change in cell polarisation towards a reduced pro-inflammatory phenotype.

**Conclusions:**

Advanced quantitative proteomics in conjunction with functional pathway network analysis is a useful tool to investigate the molecular pathways involved in inflammation.

**Supplementary Information:**

The online version contains supplementary material available at 10.1186/s12950-022-00309-8.

## Background

Inflammation is a protective response to infection or injury and an essential process in the maintenance of tissue homeostasis. It is a highly complex and tightly regulated sequence of events that involves a wide range of immune cells [1]. Macrophages play a central role in inflammation by eliciting a number of functions, including the production of a variety of inflammatory mediators (e.g., cytokines and reactive oxygen species; ROS) that help neutralize and eliminate invading pathogens and repair tissues damaged by trauma [2]. However, unregulated release of these mediators can exacerbate tissue damage in the acute phase, while failure to effectively resolve the inflammatory response can result in chronic inflammation, which is a key underlying factor in the progression of many prevalent diseases, including asthma, chronic obstructive pulmonary disease (COPD), atherosclerosis and diabetes.

Macrophages also produce eicosanoids, a group of bioactive lipid mediators including prostaglandins, leukotrienes and hydroxyeicosatraenoic acids (HETEs) that drive the progression of inflammation [3]. The ability to examine eicosanoid metabolism is critical to understanding the mechanisms associated with macrophage activation and may provide additional perspectives on inflammatory responses in conditions characterized by excessive inflammation. Proteomics is a powerful experimental approach for in-depth analysis of the protein components of cellular systems, which researchers are increasingly using as a tool to explore the biology of macrophages and molecular pathways involved in inflammatory responses. The elegant paper by Sabido and colleagues [4] details a targeted proteomic analysis to investigate the temporal changes in the expression of eicosanoid biosynthetic enzymes of the cyclooxygenase, lipoxygenase and cytochrome P450 pathways and demonstrates that a complex network of protein activity takes places following eicosanoid stimulation. Furthermore, label-free approaches [5–7] and chemical mass tagging [8, 9] have also been used in order to encompass a more global view of protein expression in lipopolysaccharide (LPS) stimulated macrophages.

In this study we have used macrophage models and applied a proteomics strategy based on stable isotope labelling with amino acids in cell culture (SILAC) to quantify the changes in macrophage protein abundance following activation, with a key focus on eicosanoid metabolism and regulation. We have used RAW 264.7 cells, a macrophage-like mouse cell line that has been widely utilized as an in vitro model to study inflammation because it exhibits many of the functional characteristics of primary macrophages [10]. The cells were exposed to physiologically relevant immune modulators that not only mimic bacterial infection but also reflect the signals produced by damaged or dying cells during clearance of dead cells and debris. The purified LPS sub-structure; 3-deoxy-d-manno-octulosonic acid–lipid A (Kdo2-lipid A/KLA) was incorporated in this study. Kdo2-lipid A shares homology with LPS, with the variable carbohydrate chains removed, ensuring consistent chemical structure and activity. Kdo2-lipid A binds to toll-like receptor 4 (TLR-4) and has equal endotoxin activity to LPS [11] whilst avoiding the large size and heterogeneity of LPS [12]. TLR4 stimulation on macrophages activates intracellular signalling pathways, including mitogen-activated protein kinases (MAPK) and nuclear factor-κ B (NF-kB), which ultimately lead to cPLA_2_ activation and prostaglandin synthesis [13]. This study takes a global approach by monitoring the protein response downstream of eicosanoid production.

Importantly, the use of SILAC has afforded key analytical advantages. Since samples are mixed prior to protein extraction, the subsequent preparation steps are identical for both labelled and unlabelled samples, thereby minimizing variability and permitting superior quantitative analysis of the cellular proteome between experimental conditions [14]. Gene ontology analysis and network mapping have been carried out to integrate the proteomic datasets and present the associated gene product attributes in terms of their cellular localisation, molecular function, biological process and pathway designation. The obtained networks and individual proteins involved have been combined with an in-depth review of the literature to allow a critical interpretation of the networks. Taken together, this work provides a holistic overview of the inflammatory response at the protein level.

## Results

### Activation of RAW 264.7 cells

RAW 264.7 cells were initially activated with Kdo2-lipid A which reflects the pathogen-associated molecular patterns (PAMPs) of gram-negative bacteria. The cells were also co-treated with ATP to mimic damage-associated molecular patterns (DAMPs) that initiate non-infectious (or sterile), pro-inflammatory responses [15]. The combination of [Kdo2-lipid A + ATP] elicits a synergistic effect in macrophages [4]. Tumor necrosis factor (TNF) is an important inflammatory cytokine that is elevated in many pathological conditions, including chronic inflammation [16]. Prostaglandins, a class of eicosanoids, are potent lipid mediators with roles in the regulation of inflammation and implications in disease [17]. As such, measurements of TNF and prostaglandins have been used in this study to support the inflammatory activation of RAW 264.7 cells.

**Fig. 1:**
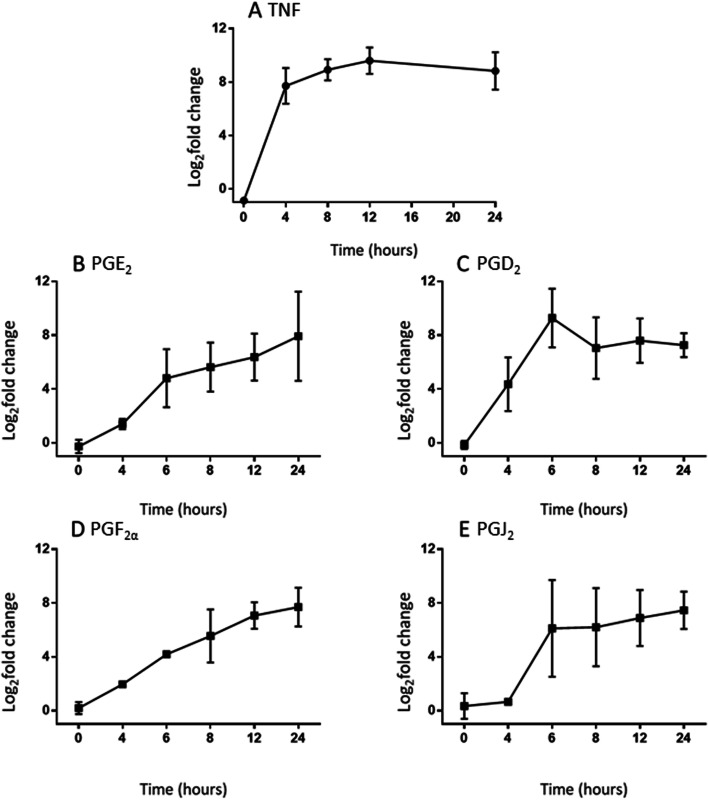
Secretion of inflammatory mediators. The log2-fold changes between PBS vehicle control and [Kdo2-lipid A + ATP] treated RAW264.7 cells in TNF (**A**) PGE_2_ (**B**), PGD_2_ (**C**) PGF_2α_ (**D**) and PGJ_2_ (**E**) release are displayed. 100 ng/ml Kdo2 lipid A treatment commenced at t = 0, 2 mM ATP treatment began at t = 4 h. The mean of 3 separate experiments (in duplicate for TNF) are shown ± SD

We observed that following stimulation, TNF and PGE_2_, PGD_2_, PGF_2α_ and PGJ_2_ were secreted into culture media over time (Fig. 1). The concentrations of TNF and prostaglandins began to plateau at 4 h post-ATP treatment, a finding consistent with previous studies which propose that 8 h post-Kdo2-lipid A treatment is near the point of maximal rate of eicosanoid production [18]. As a result, this time point was selected for the SILAC-based proteomic analyses. Of note, at t = 4 h (point of ATP addition), TNF concentrations appears to be approaching their peak, whilst PGE_2_, PGD_2_, PGF_2α_ but continue to increase in concentration and PGJ_2_ was not detected to have risen at this stage. At the chosen time point of the proteomic analysis, t = 8 h (4 h post-ATP treatment), TNF and PGD_2_ concentrations appear to have peaked whilst PGE_2,_ PGF_2α_ and PGJ_2_ appear to be continuing to increase steadily_._

### Quantitative proteomics

The SILAC dataset consisted of 4249 peptides, which corresponded to 476 proteins. Of the identified labelled and unlabelled peptide pairs, 220 were significantly different (*p* < 0.05) in their normalized abundance between control and treated cells. This equates to 136 proteins, with 48 showing increased expression following activation, whilst 88 decreased. To improve reliability, single peptide containing proteins were removed from the data set and only proteins with a fold change > 2 and < 0.5 were included, leaving 37 differentially expressed proteins, 10 of which were up-regulated and 27 down-regulated (Supplementary Fig. 1A and B and Supplementary Table 1). Furthermore, the numbers of proteins identified is consistent with previous SILAC analysis on LPS stimulated RAW 264.7 cells [19].

### Gene ontology analysis

**Fig. 2:**
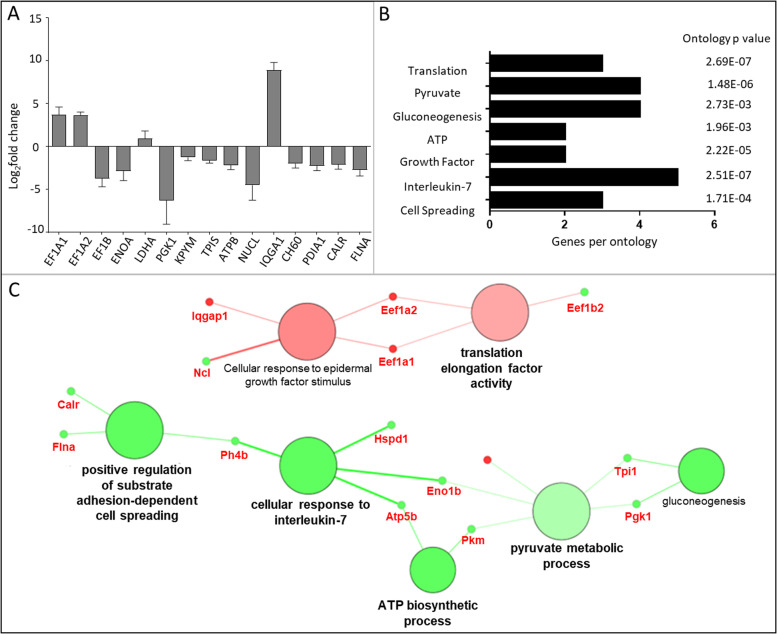
Molecular, biological and cellular component ontology network. **A** The log2-fold changes (*n* = 3 ± SD) in protein abundance between PBS control and [Kdo2-lipid A + ATP] treated RAW264.7 cells following SILAC are presented. **B** The number of identified genes per term along with associated term p-value corrected with Bonferroni step down is shown. **C** The cellular components network of proteins with a significant fold change lower 0.5 (green) or higher than 2 (red) in relative abundance after stimulation with [Kdo2-lipid A + ATP] compared to a matched PBS control. Nodes (large circles) are linked by their common genes (red labels) based on a kappa score (≥ 0.4), with the label of the most significant terms displayed. Colour intensity of nodes represent significance of ontology. Gene fusion used

Gene ontology analysis (Cystoscope) was carried out to integrate the SILAC data set and present the associated gene and gene product attributes. Three aspects of gene functions were included: molecular functions, cellular component and biological processes using gene fusion (Fig. 2). Identification of the various networks allows for a more comprehensive biological picture to be built [20]. A decrease in the GO term ‘positive regulation of substrate adhesion-dependent cell spreading’ was reported in this network, associated with a decrease in the proteins filamin A (FLNA/Flna), calreticulin (CALR/Calr) and prolyl 4-hydroxylase beta polypeptide (P4HB/P4hb). The GO term ‘cellular response to IL-7’ had also decreased, contributed to by PH4B, CH60 (HSPD1/Hspd1), Enolase 1 (ENOA/Eno1b) and ATP synthase subunit-β (ATP5B/Atp5b). Changes in energy metabolism was also suggested to be implicated in this response, with a decrease in the GO term ‘ATP biosynthetic process’, via ATP5B and pyruvate kinase (PKM/Pkm), gluconeogenesis, via triosephosphate isomerase (TPIS/Tpi1) and phosphoglycerate kinase 1 (PGK1/pgk1) and ‘pyruvate metabolic process’, via PKM, ENOA, PGK1, TPIS and lactate dehydrogenase (LDH/Ldha). The glycolytic enzymes decreased in abundance, with the exception of LDH which increased.

The ontology relating to ‘translation elongation factors (EF) activity’ increased, supported by increase in the elongation factors EF1A1 (Eef1a1) and EF1A2 (Eef1a2), whilst EEF1B2 (Eef1b2) decreased in abundance. ‘Cellular response to epidermal growth factor stimulus’ also increased and the elongation factors, IQ motif-containing GTPase-activating protein (IQGAP/Iqgap1) and nucleolin (NCL/Ncl) contribute to this.

### KEGG orthology database search

**Fig. 3:**
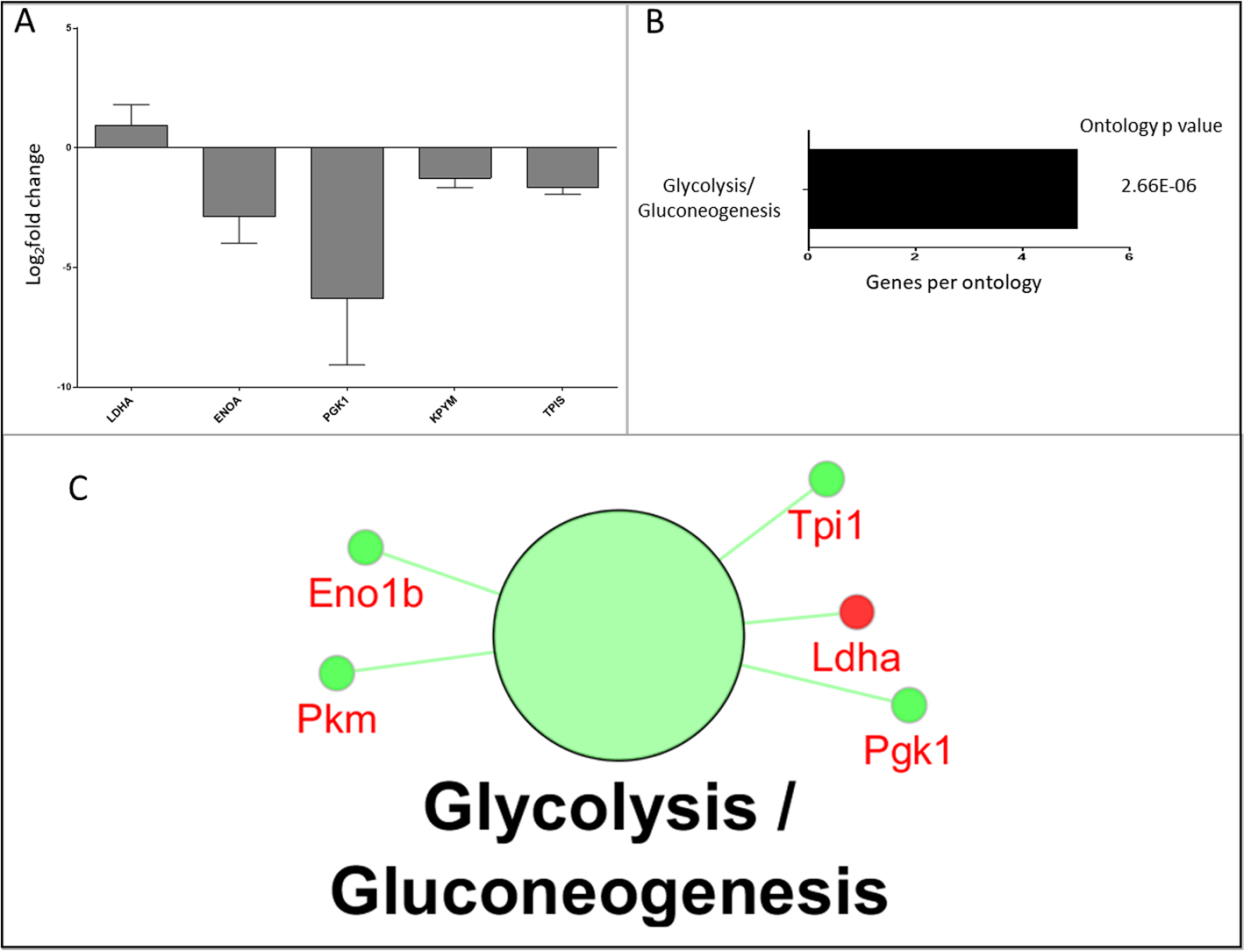
KEGG orthological network. **A** The log2-fold changes (*n* = 3 ± SD) in protein abundance between PBS control and [Kdo2-lipid A + ATP] treated RAW264.7 cells following SILAC are presented. **B** The number of identified genes per term, along with associated term *p-*value corrected with Bonferroni step down is shown. **C** The network of KEGG orthologies associated with the proteins with a significant fold change lower 0.5 (green) or higher than than 2 (red) in relative abundance after stimulation with [Kdo2-lipid A + ATP] compared to a matched PBS control. Nodes (large circles) are linked by their common genes (red labels) based on a kappa score (≥ 0.4), with the label of the most significant terms displayed. Colour intensity of nodes represent significance of ontology

The Kyoto Encyclopedia of Genes and Genomes (KEGG) search (Fig. 3 and Supplementary Fig. 2), conducted on the experimentally identified proteins, identified a decrease in glycolysis/gluconeogenesis, supporting the biological and molecular functional network data. The associated proteins included TPIS, PGK1, ENOA, PKM and LDH. The KEGG pathway highlights how the proteins that decrease in abundance are involved in the progression of glycolysis, whilst LDH—which increases—is involved in an alternative (anaerobic) pathway. Furthermore, the significantly altered proteins all form part of the lower glycolytic ‘trunk’ pathway, which is the section of the pathway which follows fructose-1–6-p up to lactose (Supplementary Fig. 2).

### Reactome database search

**Fig. 4:**
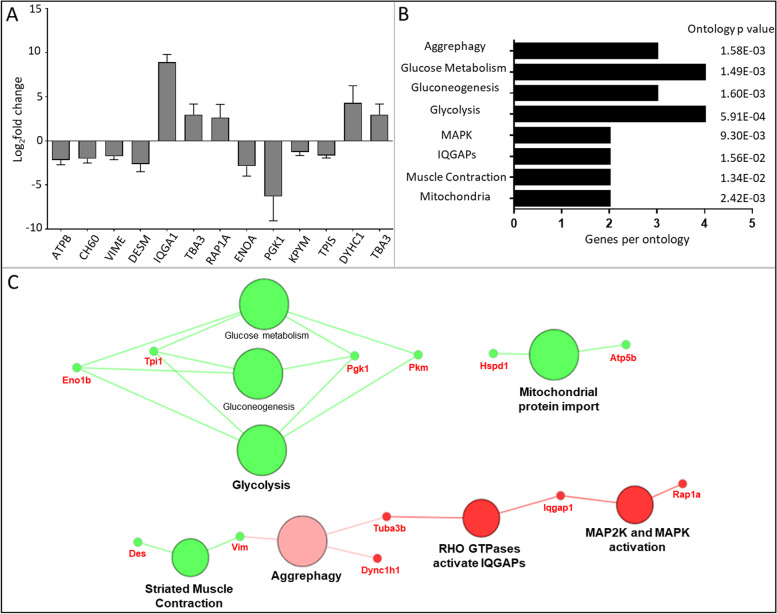
Reactome Pathways network. **A** The log2-fold changes (*n* = 3 ± SD) in protein abundance between PBS control and [Kdo2-lipid A + ATP] treated RAW264.7 cells following SILAC are presented. **B** The number of identified genes per term along with associated term *p*-value corrected with Bonferroni step down is shown. **C** The network of Reactome pathways orthologies associated with the proteins with a significant fold change lower 0.5 (green) or higher than 2 (red) in relative abundance after stimulation with [Kdo2-lipid A + ATP] compared to a matched PBS control. Nodes (large circles) are linked by their common genes (red labels) based on a kappa score (≥ 0.4), with the label of the most significant terms displayed. Colour intensity of nodes represent significance of ontology

**Fig. 5:**
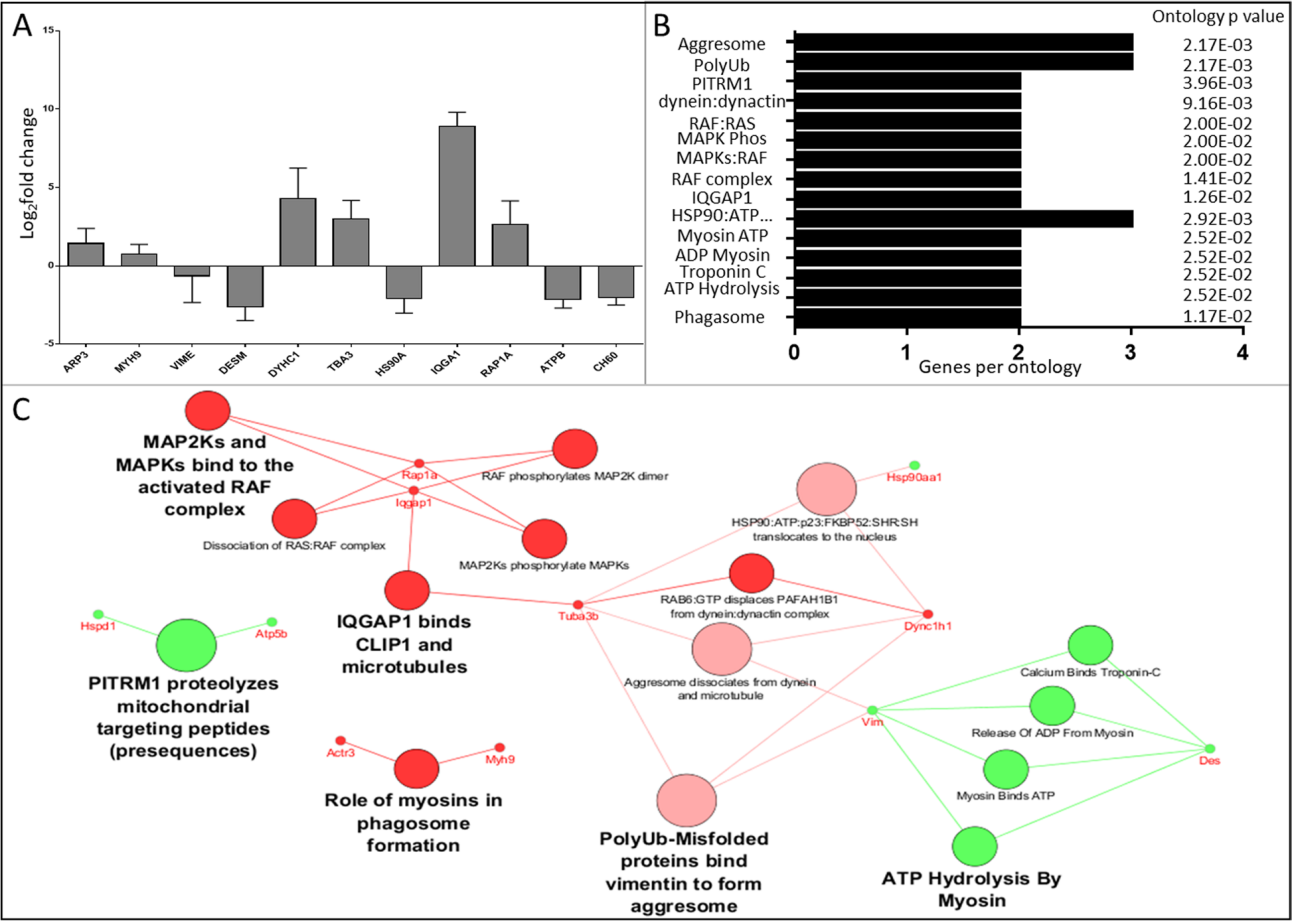
Reactome Reactions network. **A** The log2-fold changes (*n* = 3 ± SD) in protein abundance between PBS control and [Kdo2-lipid A + ATP] treated RAW264.7 cells following SILAC are presented. **B** The number of identified genes per term along with associated term *p*-value corrected with Bonferroni step down is shown. **C** The network of Reactome reactions orthologies associated with the proteins with a significant fold change lower 0.5 (green) or higher than 2 (red) in relative abundance after stimulation with [Kdo2-lipid A + ATP] compared to a matched PBS control. Nodes (large circles) are linked by their common genes (red labels) based on a kappa score (≥ 0.4), with the label of the most significant terms displayed. Colour intensity of nodes represent significance of ontology

The Reactome Database was searched for pathways (Fig. 4) and reactions (Fig. 5) using the experimentally identified proteins. This database includes molecular events (reactions) which are organized into cellular pathways and supports joining and organizing of reactions to describe the molecular basis of an entire biological process [21]. Pathways which decreased in the Reactome Pathways network included glycolysis, glucose metabolism and gluconeogenesis, which support the KEGG and gene ontology network analysis. In addition, mitochondrial protein import decreased, supported by decreases in HSPD1 and ATP5B, and ‘striated muscle contraction’ decreased, supported by decreases in the proteins desmin (DESM/Des) and vimentin (VIME/Vim). ‘Aggrephagy’, ‘RHO GTPases activate IQGAPs and ‘MAP2’ and MAPK activation’ were shown to increase. A decrease in the abundance of VIME and increases in tubulin (TBA3/Tuba3b) and cytoplasmic dynein 1 (DYHC1/Dync1h1) contribute to the Aggrephagy ontology, increases in IQGAP and Ras-related protein Rap-1A (RAP1A/Rap1a) contribute to MAPK ontology, whilst increases in TBA3 and IQGAP contribute to the IQGAP activation ontology.

The Reactome Reactions ontology search identified decreases in ‘PITRM proteolyzes mitochondrial targeting peptides’, supported by decreases in HSPD1 and ATP5B. ‘ATP hydrolysis by myosin’ and ‘myosin binds ATP’ were also indicated to decrease, supported by decreases in VIME and DESM. The increases in this network included ‘MAP2Ks and MAPKs bind to the activated RAF complex’, ‘IQGAP1 binds CLIP1 and microtubules’, and ‘PolyUb microfolded proteins binds vimentin to form aggresomes’. These ontologies support the significant ontologies highlighted in the Reactome Pathways network. Furthermore, an increase in phagosome formation was indicated, contributed by actin (ARP3/Actr3) and myosin (MYH9/Myh9).

### Proteins of eicosanoid metabolism and regulation

**Fig. 6:**
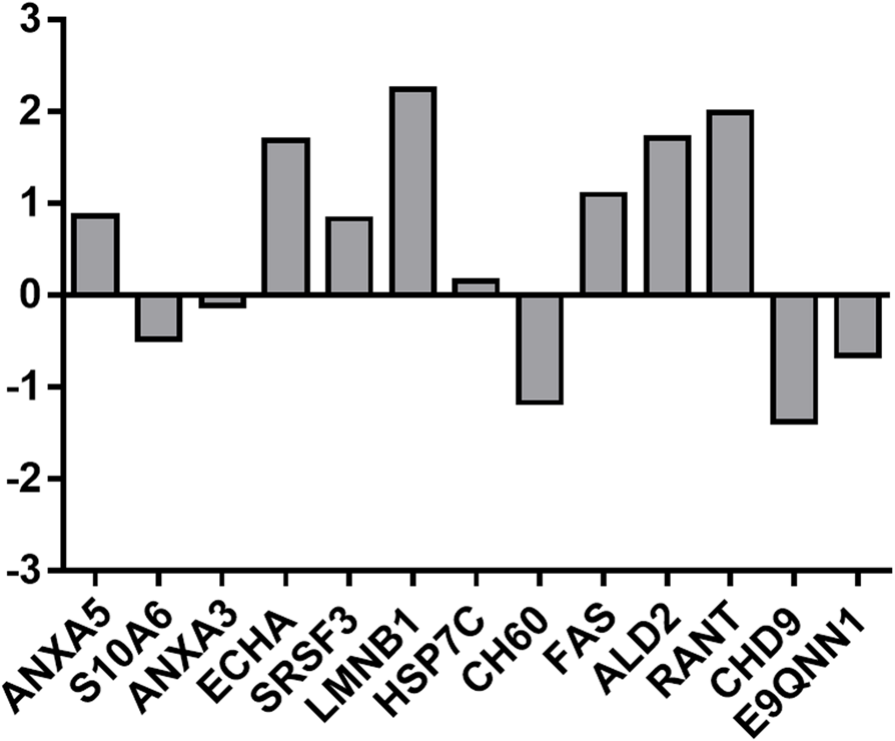
Changes in Eicosanoid Related Proteins. The log_2_-fold changes in protein abundance between PBS control and [Kdo2-lipid A + ATP] treated RAW 264.7 cells are shown. Proteins COX5A, PTGR1 and HMGB1 were not present in vehicle control therefore log fold change cannot be obtained

It is often challenging to detect eicosanoid-related proteins in global proteomic screens due to their relatively low abundance and transient expression. Indeed, in our initial search using Progenesis QIP in conjunction with MASCOT, we did not observe these proteins. However, the use of multiple data capture platforms is able to provide a consensus of protein identification and reduce key sources of error in proteomics data processing [22]. We therefore performed a subsequent search using an alternative platform, namely Proteome Discoverer in conjunction with SEQUEST, and 16 proteins that have ontologies directly linked to eicosanoid pathways were identified, including those with direct involvement in lipid and eicosanoid metabolism and TLR4 activation (Fig. 6 and Supplemental Table 2).

## Discussion

### Inflammatory mediator production

Most eicosanoids act through G protein-coupled receptors (GPCRs), resulting in activation of the cyclic adenosine monophosphate (cAMP) or the phosphatidylinositol pathways, and calcium signalling, triggering a widespread variety of effects, including the activation of inflammation [23]. The prostaglandin receptors include the PGD receptor, DP; the PGE receptors, EP1, EP2, EP3, and EP4; the PGF receptor, FP; the PGI receptor, IP; and the thromboxane A receptor, TP. In addition, some eicosanoids, including the cyclopentane prostaglandins, act through peroxisome proliferator-activated receptors (PPAR) that function as transcription factors [24]. PGD_2_ is dehydrated to the cyclopentenone prostaglandin PGJ_2_, which is further metabolised to derivatives including 15-deoxy-delta12-14-prostaglandin J_2_ (15-d-PGJ_2_), which acts as the endogenous ligand on the PPARg and PGD_2_ receptors [25]. Prostaglandins are generally considered to be pro-inflammatory, but some have also been associated with anti-inflammatory and pro-resolving roles, including PGD_2_ [26], PGE_2_ [27] and particularly PGJ_2_ via 15-d-PGJ_2_ activation of PPARg [28]. Ultimately, the identified measurements of TNF and prostaglandins (Fig. 1) indicate a successful activation of the inflammatory response and support the selection of the chosen timepoint (t = 8 h) for SILAC-proteomic analysis.

### Macrophage activation

Gene ontology analysis, on the experimentally identified proteins, identified a decrease in positive regulation of substrate adhesion-dependent cell spreading. Macrophages have the ability to polarize between different phenotypes that are associated with different functional activities, such as proliferation and apoptosis. Inactive macrophages (M0) can be polarised towards classically activated (M1) or alternatively activated (M2) subsets, although cells can exhibit characteristics of both subsets. Polarisation alters macrophage morphology (round or spindle like, with M2 cells exhibiting an elongated shape with more spindles compared to M1 subtypes) and proteomic profiles, which reflect pro- and anti-inflammatory functions of M1 and M2 cells respectively [29]. M1 macrophages secrete pro-inflammatory mediators, including eicosanoids, whilst eicosanoid receptor binding is associated with macrophage polarisation. PPARγ activation, following 15-d-PGJ_2_ stimulation [30] and activation of the PGE_2_ receptor EP4 [31] and the PGF_2__α_ FP receptor [32], promote M2 polarisation of macrophages and anti-inflammatory functions. Therefore, it is possible that the measured increases in prostaglandins are inherently linked to this ontology, depending on activation of the target receptors. Furthermore, TLR activation of macrophages, through LPS stimulation, has been shown to induce morphological changes and cell spreading via MyD88, Irak, p38, Rap1, and β2-integrins and this is vital to host defence [33]. This is a rapid response mechanism, which may explain why a decrease in this process is observed at the timepoint used in this study. This is supported in Fig. 1 where at t = 8 h, selected for the gene ontology analysis, some of the inflammatory mediators are beginning to plateau (Fig. 1A, C and E), suggesting the inflammatory response is approaching its maximum.

Decreases in FLNA and CALR are associated with a decrease in cell spreading (Fig. 2). FLNA is a scaffold protein that regulates actin polymerization. FLNA is localized to and regulates stability of podosomes which are involved in mesenchymal migration of macrophage cells. Contractility within the actin cytoskeleton via actin and myosin, is necessary for shape-induced M2 polarisation [34]. CALR is a calcium binding chaperone that is also of interest for its functions outside the endoplasmic reticulum, where it has immunomodulatory properties [35]. At the cell surface, CALR is associated with apoptotic-cell removal in a process called efferocytosis, which is important to prevent cell lysis and the release of pro-inflammatory and antigenic autoimmune components [36]. Further CALR contributes to an increase in NF-κB signalling, via IκB stabilisation [37] and facilitates the folding of major histocompatibility complex (MHC) class I molecules for presentation [38]. As such, a decrease in this protein may act to reduce these inflammatory activities. The decrease in ‘Striated muscle contraction’ in the Reactome Pathways network (Fig. 4) also highlights the changes to motility which underpin macrophage activation and polarisation. The proteins DES and VIM support this ontology. These proteins are both class-III intermediate filaments, which support cell mechanics, signalling and the maintenance of normal cell homeostasis [39].

IL-7, which as indicated to decrease in the gene ontology network (Fig. 2), is a potent inducer of M1 macrophage differentiation [40] and macrophage migration and induces TNF secretion and PI3K/AKT-dependent and -independent activation of NF-κB [41]. Blockade of IL-7 signalling has previously been shown to promote the M2 macrophage subtype in thioglycollate-elicited peritoneal macrophages [42]. Furthermore, IL-7 is also a key regulator of T-cell development and homeostasis [43] and in T-cells, IL-7 signalling is highly controlled by IL-7 itself which negatively regulates its receptor, IL-7R, and results in increased cell survival [44]. PGE_2_ has been suggested to supress IL-7-dependent T-cell expansion [45]. Therefore, it is possible that this mechanism is reflected in macrophages, resulting in a decrease in responses to IL-7 signalling. Indeed, this dynamic regulation of IL-7 signalling is also suggested in macrophages [46] but would need to be explored further. The downregulation in IL-7, suggested at the time point used in this study (t = 8 h), would suggest a promotion of the pro-resolving phenotype at the protein level and may be a response to the high TNF signalling and eicosanoid release, by way of a control mechanism to prevent excess inflammation.

Together the reduction in cell spreading and IL-7 signalling indicate that the cells have a reduced migration and pro-inflammatory phenotype. P4HB links these two processes. P4HB belongs to the protein disulfide-isomerase (PDI) family and is a key enzyme involved in the rearrangement of disulfide bonds in proteins [47]. In platelets, this protein has been associated with platelet aggregation via its regulation of NADPH oxidase (NOX) enzyme activity to promote oxygen radical production [48], central to antimicrobial activity. NOX is highly expressed in phagocytes therefore the ability of P4HB in macrophages to promote aggregation through oxidative stress in the surrounding environment should be further explored. ROS generation from macrophages has also been implicated in platelet activation via PLA2 activation, resulting in thromboxane generation. P4HB is suggested to control the localisation of various endoplasmic reticulum (ER)-located enzyme components, which is crucial for NOX complex formation at the cell membrane [49]. P4HB acts as an ER chaperone to inhibit the aggregation of misfolded proteins and is a marker of ER stress. Furthermore, TLR activation of macrophages is able to repress ER stress-induced mRNAs, including the production of PDI mRNA [50]. In support of these findings, we found that this protein was decreased, indicating a reduction in disulfide bond rearrangement or ER stress. Previous GeLC-MS^n^ analysis identified a decrease in PDI abundance in 48 h LPS-stimulated monocytes, which is suggested to be due to a promotion of proteolytic degradation of PDI via oxidative stress [51, 52]. As such, the identified decrease in P4HB may reflect a feedback mechanism to control and prevent continued ROS production after the initial respiratory burst, possible to prevent chronic damage to tissue. However, the complete mechanism of the regulation of NOX enzymes by PDI is unclear, and modifications, such as oxidation and glycosylation, as well as calcium signalling are likely to be key [48, 53]. Therefore, protein abundance alone does not reflect the activity of this particular pathway.

CH60 also contributes to the IL-7 signalling ontology (Fig. 2). CH60 is a molecular chaperone for folding and assembly of proteins in ER and is associated with pro-inflammatory response and macrophage activation via a promotion of NF-κB and therefore IL-6 and TNF expression [54]. The identified decrease in abundance of this protein, at t = 8 h, therefore suggests a feedback mechanism at the protein level to counteract the increased release of TNF which the ELISA cytokine assay identified (Fig. 1A).

IQGAPs and MAPK signalling was indicated to be altered in both Reactome networks (Fig. 4 and 5), exploited at the protein level. IQGAP constitute a family of scaffold proteins. IQGAP is mainly associated with actin filament and has been implicated in the control of cellular motility and morphogenesis through regulation of actin organization, cell–cell adhesion, microtubule stability and small G proteins [55]. RHO GTPases activate IQGAPs when bound to GTP and play critical roles in macrophage function including actin-based cytoskeletal rearrangements for cell adhesion, spreading and motility, and pathogen responses [56]. Pertinent to this study, IQGAP can act as a scaffold protein in MAPK signalling and can provide a feedback loop to control MAPK signalling [57]. The MAPK signalling pathway involves a cascade of phosphorylation events and regulates a wide variety of cellular functions including transcription, cell proliferation, differentiation, stress responses, movement, cell division and apoptosis [58].

Ligand binding to GPCR eicosanoid receptors regulates MAPK signalling (directly or indirectly), resulting in diverse biological functions depending on the receptor and activities of the G protein components (G_i_, G_s_ or G_q_). For example, PGE_2_ binding to EP1 receptor results in G_q_ activation and p38 MAP kinase-dependent IL-10 production, whilst PGE_2_ binding to the EP2 receptor results in G_s_ activation, cAMP production and inhibition of the MAPKK activator, Raf [59]. Furthermore, MAPK signalling can suppress PPARγ activity, by inhibitory phosphorylation or nucleo-cytoplasmic compartmentalization, which in turn downregulates pro-inflammatory responses, suggesting a regulatory mechanism [60]. The increase in prostaglandins may therefore contribute to the regulation of MAPK signalling, and MAPK signalling may in turn contribute to the regulation of prostaglandin production.

Previously, TLR4 activation has been shown to active MAPK signalling pathways via MAL/MyD88 and TRAM/TRIF mediated signalling [61, 62]. However, these proteins are kinases and are activated via phosphorylation and deactivated by phosphatases; studies have shown that changes in MAPK gene expression do not generally corelate with phosphorylation [63]. Therefore, the protein abundances provided in these results do not reflect protein activity. However, they do indicate availability and as such, the decreases in abundance may reflect a promotion of degradation of these proteins, at the selected timepoint, as a form of a counter-regulation in the face of increased activation. RAP1A, which contributes to this ontology term, is a GTPase associated with promotion of macrophage phagocytosis by promoting of complement induced opsonisation [64].

Aggrephagy was suggested to increase in the Reactome Pathways search (Fig. 4). Aggrephagy is the sequestration of aggregated proteins into aggresomes which recruit chaperones and proteasomes for selective protein autophagic degradation. Autophagy is thought to be a key regulator of macrophage functions such as the control of monocyte differentiation and prevention of default apoptosis. LPS promotes autophagy through MAPK signalling [65]. In addition, TLR4 activation triggers the NF-kB and MAPK signalling pathway, which is consistent with this ontology. Therefore, in this Reactome network we see an increase in aggrephagy which recycles materials and decreases mitochondrial activity, promoting catabolism. PPAR signalling modulates autophagy with PPARg inhibiting autophagy in macrophages [66], whilst cPLA_2_ and some metabolites, including PGD_2_, have been shown to induce autophagy [67]. This is not the case for all cPLA_2_ metabolites, as EP4 activation inhibits excessive mitochondrial autophagy and subsequent loss of mitochondria, which helps to maintain the energy balance within cells [68]. It is plausible that the measured increase in prostaglandins, namely PGD_2,_ contributes to the increase in autophagy at the selected timepoint of proteomic analysis.

DYHC1 and TBA3, which contribute to the aggrephagy related ontology, associate with microtubules and rearrangement of the microtubule cytoskeleton is crucial for the regulation of immune responses, including cell morphology, motility, division, and intracellular organization and transport. Previously, IFN-γ and LPS stimulation of RAW 264.7 macrophages have been shown to induce an increase in stabilized cytoplasmic microtubules and western blot and global proteomic analysis of IFN-γ and LPS stimulation of RAW 264.7 macrophages identified increases in the abundances, co-localisation of microtubule-associated proteins including dynein and tubulin. These changes in microtubule proteins were accompanied by phenotypic changes in activated macrophages including changes in cell size and cell spreading, highlighting the importance of the microtubule network in activated macrophages [69].

Phagocytosis was also suggested to decrease in the Reactome Reactions search (Fig. 5). Eicosanoids can contribute to the role of macrophages as professional phagocytes. For example, activation of the PGE_2_ EP2 and EP3 receptors suppress phagocytosis via cAMP stimulation in alveolar macrophages [70]. By contrast, PPARγ promotes phagocytosis [71] and macrophage recognition of apoptotic cells [72]. Phagocytosis is a dynamic process requiring macrophages to undergo membrane and cytoskeleton remodelling. Membrane tension supports this process; initially pseudopods extend aided by actin polymerisation and following this membrane tension promotes remodelling and vesicle exocytosis promoting phagocytosis. The decrease in ATP and myosin interaction, also in the Reactome network, indicates a downregulation in the mechanical changes of the macrophage cells such as a decrease in ECM degradation and membrane tension. This may indicate a reduction in macrophage polarisation and phagocytosis and potentially a shift towards the resolution phase at the selected timepoint.

Epidermal growth factor stimulus was suggested to increase in the gene ontology network (Fig. 2), exploited at the protein level, and the elongation factors, IQGAP and nucelolin contribute to this. Nucleolin is a multifunctional RNA-binding nuclear protein, previously suggested to negatively regulate pro-inflammatory functions in macrophages, including inflammatory gene regulation [73]. Furthermore, nucleolin is suggested to negatively regulate macrophage foam cell formation by reducing lipid accumulation and oxidative stress [74]. As such the observed increase in the expression of nucleolin, at the selected timepoint, may be a result of a feedback mechanism to reduce inflammatory functions of macrophages.

### Metabolic regulation

All networks, which were analysed at the protein level, identified aberrated energy metabolism. Metabolic reprogramming of macrophages similar to the Warburg effect occurs during LPS stimulated inflammatory responses, with M1 macrophages displaying an increase in glycolysis and M2 macrophages favouring oxidative phosphorylation. Indeed, LPS induction of macrophages has been shown to induce PKM2 expression, which alters metabolism in activated macrophage cells and promotes a shift towards the M2 subtypes [75]. Mitochondrial Ca^2+^-independent PLA_2_ (iPLA2) influences fatty acid β-oxidation, oxygen consumption, energy expenditure [76]. Furthermore, PPARγ also promotes an increase in mitochondrial mass and thus ATP biosynthesis [77] and is required for stimulation of macrophage respiration and promotion of the alternative phenotype, through changes in metabolic regulation, namely glutamine metabolism [75, 78]. Similarly, PGE_2,_ thus PGE_2_ receptor activation, promotes oxidative phosphorylation and M2 polarization [76, 79] and downregulates the mitochondrial membrane potential, via cAMP, which is linked to oxidative phosphorylation, providing the motive force for ATP synthesis [75, 80]. The eicosanoid response is therefore coupled to metabolic regulation and macrophage polarisation of macrophages.

The decrease in ATP biosynthetic process may indicate that ADP is in short supply in stimulated RAW 264.7 macrophage cells, at the selected timepoint, possibly due to the high energy demand of the inflammatory response or indicating that the cells are in an early stage of apoptosis, in which mitochondrial dysfunction is central [81, 82]. PKM, which was decreased, is a glycolytic enzyme that catalyses the transfer of a phosphoryl group from phosphoenolpyruvate to ADP, generating ATP. Indeed, pyruvate is a pivotal component of energy metabolism. The corresponding decrease in the metabolism of pyruvate reflects the reduction in glycolysis/gluconeogenesis also observed in this network and in the KEGG and Reactome networks.

ATP synthase, ATP5B, which contributes to the production of ATP from ADP in the respiratory chain, was found to decrease. ATP5B is a subunit of mitochondrial ATP synthase (complex-V) for production of ATP from ADP in the respiratory chain and maintenance of energy homeostasis. ATP5B is increased via advanced glycation end products (AGE) and ROS production [83]. Together, the decrease in abundance of ATP5B and the glycolytic proteins indicates mitochondrial dysfunction, possible due to the promotion of apoptosis, or suggesting a decrease in the respiratory chain and ROS production at the selected timepoint.

Changes to mitochondrial activity was indicated in the Reactome Database search with a decrease in mitochondrial targeting peptides. Mitochondria in mammalian cells contain around 1,500 proteins [84]. Most of these proteins are encoded by nuclear genes, synthesized on cytosolic ribososmes and imported into the mitochondria. Mitochondria are critical for ATP generation, driving metabolic processes, including the biosynthesis of amino acids and lipids, as well as playing a key role in cellular signalling pathways and the activation of apoptosis. The decrease in this ontology indicates a decrease in mitochondrial activity, which corroborates the findings from the other networks, that activated macrophages undergo metabolic reprogramming. PITRM is also known as mitochondrial presequence protease (PreP) and metalloprotease 1 (MTP-1). PITRM facilitates proteostasis and is associated with extracellular matrix degradation and the modification of proteins such as cytokines and chemokines with important roles in the destruction of atherosclerotic plaques. The metalloprotease function is associated with NF-κB activity and the prostaglandin-cAMP pathway. LPS-mediated stimulation of monocytes has previously been reported to induce metalloprotease production in a dose dependent manner and this effect was prevented by PGE_2_ inhibition [85].

LDH is a key enzyme catalysing the interconversion between lactate and pyruvate and coupling the interconversion of NAD^+^ and NADH. When pyruvate is in low abundance, LDH regenerates NAD^+^ for anaerobic glycolysis to provide short term energy supply. Our results show that PKM, which catalyses the production of pyruvate, decreases while LDH increases, indicating that anaerobic glycolysis is taking place (Fig. 2 and 3). Enolase is multifunctional and not only plays a role in glucose metabolism but also acts as a plasminogen receptor which contributes to macrophage function [86] and participates in cellular stress responses via promoting hypoxic tolerance [87]. Enolase has also been shown to induce macrophage activation and cytokine secretion [88]. The decrease in ENOA therefore is not only associated with a decrease in glycolysis, but also a decrease in cellular responses to interleukin 7, which promotes B and T cell activity and the secretion of inflammatory cytokines. Phosphoglycerate kinase 1 (PGK1) can also act as a disulfide oxidoreductase and is a key enzyme involved in the generation of glycerol-3-phosphate, coupled to the conversion of ADP to ATP in glycolysis. Further, TPIS is a metabolic enzyme that contributes to D-glyceraldehyde-3-phosphate (G3P).

### Protein and gene expression

Translation elongation factors act on the ribosome to assist in elongation of the polypeptide chain and therefore promote protein synthesis. EF1A (EF1A1 and EF1A2) acts first to deliver amino-acylated tRNAs to the ribosome in a GTP-dependent reaction, followed by EF1B which functions as a nucleotide exchange factor, recycling EF1A into its active GTP form. Finally, EF2 catalyses the GTP-dependent translocation step of each codon of RNA along the ribosome [89]. Therefore, the increase in translation elongation factor activity indicates an increase in protein synthesis at the selected time point (t = 8 h). EF1A1 and EF1A2 are the two isoforms of EF1A which is a highly abundant protein associated with many additional cellular processes including cell growth, proliferation, apoptosis and cytoskeletal organisation [90]. Whilst EEF1A is the ubiquitously expressed isoform, EF1A2 is overexpressed in tumours and is associated with anti-apoptotic activities and terminal differentiation. The increase in EF1A2 suggest an induction of cellular processes to help adapt to the inflammatory response. This elongation factor has previously been associated with TNF translation elongation [91].

Protein kinase C (PKC), a key stimulator of cell proliferation and differentiation, has been shown to upregulate both transcription and translation rates via its kinase activity. PKC promotes activity of elongation factors [92]. PPARg binding to 15-d-PGJ2 inhibits PKC signalling, promoting anti-inflammatory properties [93]. Furthermore, activation of many eicosanoids receptors, such as EP1, results in PKC signalling [94]. Therefore, the release of eicosanoids may contribute to the suggested regulation of elongation factors.

### Proteins of eicosanoid metabolism and regulation

A further 16 proteins that have ontologies directly linked to eicosanoid pathways were identified (Fig. 6). Calcium binding proteins, including the annexins (ANXA5 and ANXA3) and protein S100-A6 (calcyclin) are known to play an upstream role in the regulation of prostaglandin synthesis [95]. Annexin A5 is involved in apoptosis and downregulation of the phosphatidylinositol 3-kinase (PI3K)/Akt/NF-kB signalling pathway in certain tumours [96] and has been demonstrated to inhibit cPLA_2_ in peripheral blood lymphocyte cell lines [97]. These reports suggest annexin A5 may have immunosuppressive roles and downregulate the synthesis of eicosanoids. Furthermore, annexin A5 is suggested to interact with pyruvate kinase 2, leading to metabolic reprogramming from glycolysis to oxidative phosphorylation in macrophages [98]. Anenxin-V was found to be elevated and may be linked to the metabolic reprogramming highlighted in the functional network analysis. Annexin A3 is involved in macrophage cell migration, inhibition of prostaglandin synthesis via suppression of PLA_2_ and promotion of inflammatory resolution [99]. However, this mediator did not appear to be altered in our study. S100-A6 regulates immune homeostasis and is suggested to function as DAMP to activate p38 and JNK downstream of the TLR4 and p65 pathways [100], therefore may promote inflammation and prostaglandin production. This protein was found to be decreased, suggesting a downregulation of the pro-inflamamtory response.

Heat shock proteins (HSP7C and CH60) are a family of molecular chaperones that play an important role in protein refolding. These proteins have been linked to prostaglandin and LPS receptor binding [101], which is pertinent to this study. HSP7C did not appear to be altered, whilst CH60 was found to be decreased. HSP7C is constitutively and abundantly expressed [102], which may explain why this protein did not appear to change. CH60 contributes to the IL-7 signalling ontology (Fig. 2) and in addition to the promotion of NF-kB, stimulates human monocytes via a CD14/TLR2/TLR4-dependent mechanism, promoting the release of pro-inflammatory cytokines [103]. By contrast, HSP7C is associated with anti-inflammatory actions and downregulation of LPS responses including NF-kB, TNF, IL-6, NO, iNOS and COX-2 [104], suggesting an immunosuppressive mechanism.

Serine/arginine-rich splicing factor 3 (SRSF3) has been shown to regulate inflammatory responses by repressing translation of inflammatory genes in microglia, including LPS response genes [105]. Therefore SRSF3 – which was increased – may suppress pro-inflammatory responses upstream of prostaglandin production. Lamin-B1 (LMNB1) was also increased. Previously, LMNB1 mRNA was increased following IL-10 stimulation of rheumatoid arthritis synovial macrophages, suggesting a possible anti-inflammatory role [106], a feature that has been supported in other models [107]. Both SRSF3 and LMNB1 are also associated with JNK dependent inositide-dependent phospholipase Cb1 (PI-PLC b1) nuclear functions, including cell growth, proliferation, and differentiation [108].

Aldose reductase promotes inflammatory activation by mediating LPS-induced signalling including activation of PKC and phospholipase C (PLC), nuclear translocation of NF-kB, and phosphorylation and proteolytic degradation of IkBa in macrophages [109]. In human endometrial cells, aldose reductase functions as a synthase for PGF_2α_ production, and subsequently PGE_2,_ via a positive feedback loop [110]. Therefore, the identified increase in aldose reductase-related protein 2 (ALD2) may be associated with the continued increase in PGF_2__α_ (and PGE_2_) throughout the eicosanoid time course analysis (Fig. 1); both these mediators can promote anti-inflammatory responses. Ran has been closely associated with many inflammatory signalling pathways including the promotion of the translocation of NF-kB and TGF-b transcription factors and appears to be involved in a positive feedback loop with MAPK and PI3K [111]. Furthermore, many eicosanoid receptors are GPCRs, therefore the increase in GTP-binding nuclear protein Ran (RANT) may be associated with activation of the prostaglandin receptors.

Chromodomain-helicase-DNA-binding protein 9 (CHD9) acts as a transcriptional coactivator for PPARa which is involved in eicosanoid signalling including HETEs, epoxygenases (EETs) and dihydroxyeicosatrienoic acids (DHETs). The decrease in CHD9 suggests a downregulation on eicosanoid signalling which may have various inflammatory consequences. ATP-dependent RNA helicase A (E9QNN1) is associated with NF-kB signalling, independent of its DNA-sensing function, and is therefore suggested to have a pro-inflammatory role [112]. The decrease in E9QNN1 may be associated with a promotion of anti-inflammatory and pro-resolving activities.

Prostaglandin reductase 1 (PTGR1) is involved in metabolic inactivation of eicosanoids, including prostaglandins, leukotrienes and lipoxins, via a reductase reaction and therefore has pro-inflammatory, anti-inflammatory, and pro-resolving effects, depending on its target [113]. High mobility group protein B1 (HMBG1) has various roles, including acting as a DAMP, LPS homing to the cytosol, and signalling through TLR4 to promote production of pro-inflammatory cytokines including TNF. HMGB1 initially acts as a pro-inflammatory mediator. However, in high quantities, HMBG1 is associated with immune suppression [114]. Furthermore, HMGB1 induces the expression of 15-hydroxyprostaglandin dehydrogenase (15-PGDH), the inactivating enzyme of pro-resolving lipid mediators, thereby preventing inflammatory resolution [115]. HMGB1-mediated regulation of the expression of PTGR1 has been associated with downregulation of inflammatory resolution [116]. Both these proteins were increased in the data.

A subunit of the trifunctional enzyme (ECHA) catalyses the last three steps of mitochondrial beta-oxidation of long chain fatty acids [117]. As such, the identified increase in ECHA abundance may promote an increase in beta oxidation to replenish energy or to reduce availability of fatty acids, supressing their biological activity. Previously, LPS treatment on macrophages has been shown to enhance beta oxidation for removal of polyunsaturated fatty acids, including prostaglandins, to regulate their inflammatory function [118]. Cytochrome c oxidase subunit 5A (COX5A) is the last enzyme in the mitochondrial electron transport chain which drives oxidative phosphorylation. The increase in COX5A therefore supports the network analysis with a promotion of the M2 macrophage subset and possible promotion of inflammatory resolution.

Fatty acid synthase (FAS) is a protein complex that catalyses the formation of long-chain fatty acid (primarily palmitate). FAS has been associated with TLR4 activation and inhibition of FAS results in a reduced pro-inflammatory response in macrophage cells, including aberrated TNF production [119]. Therefore, FAS is suggested to have a pro-inflamamtory role, facilitating production of eicosanoids and the increase in FAS may be associated with the measured increase in prostaglandins. Altogether, these data provide further insights of eicosanoid-mediated responses to inflammatory stimuli and the roles of these proteins in the eicosanoid response should be investigated further.

## Conclusions

In conclusion, this research highlights the benefits of advanced quantitative proteomics in conjunction with functional pathway network analysis to investigate the molecular pathways involved in inflammation. The ability to explore eicosanoid metabolism at the protein level will be critical to understanding the role that these lipid mediators play in disease states and offers the potential to identify novel therapeutic targets for chronic inflammatory conditions. These data are similar to those from a recent label-free proteomics approach [7]. Future experiments may wish to combine numerous experimental approaches to monitor the activation state of proteins via modifications such as phosphorylation or calcium signalling, which may be confounding factors to a metabolic pathway. Furthermore, due to the highly dynamic nature of the inflammatory response, the investigation at additional time points may reveal greater insights into the associations of proteins and lipids during the progression of the inflammatory response.

## Methods

### Cell culture

RAW 264.7 mouse monocyte macrophage cells were obtained from the European Collection of Authenticated Cell Cultures (ECACC, Public Health England, Porton Down, UK) and seeded in 6 well plates at a density of 1 × 10^6^ cells per well. Cells were grown for 24 h in high glucose Dulbecco’s Modified Eagles Medium *(*DMEM, Fisher Scientific, Loughborough, UK) supplemented with 4 mM L-glutamate, 10% (v/v) heat-inactivated foetal calf serum (FCS) (Fisher Scientific) and 1% (v/v) penicillin/streptomycin (Fisher Scientific). RAW 264.7 cells were incubated with 100 ng/ml Kdo2-lipid A (Avanti Polar Lipids, Alablaster, AL, USA) for 4 h and then co-treated with 2 mM adenosine 5′-triphosphate (ATP) magnesium salt (Merck, Poole, UK) for a further 4 h [4]. Cells were harvested by gently scraping in isotonic phosphate buffered saline (PBS) pH 7.4 and pelleted by centrifugation at 5,500 × *g* for 5 min. The supernatant was removed and each cell pellet were resuspended in 150 μl water with 50 μl of 1 × Complete Protease Inhibitors (Roche, Welwyn Garden City, UK) and lysed with a QSONICA Q500 probe sonicator (Newtown, CT, USA) for 3 × 10 s pulses at 25% amplitude. The protein concentration was determined using the Coomassie Plus Protein Assay (Pierce Biotechnology, Rockford, IL, USA*).* Experiments were conducted on three separate occasions.

### Measurement of TNF

Cell-free media from control and treated cultures were collected at 0, 4, 8- and 20-h post ATP treatment. The concentrations of tumor necrosis factor (TNF) in the supernatants of RAW 264.7 cell cultures were determined using the TNF alpha Mouse Instant ELISA Kit (Invitrogen, Paisley, United Kingdom) according to the manufacturer’s instructions.

### Prostaglandin analysis

Prostaglandins were isolated from cell media. 4 ml ice cold methanol containing 1 ng PGE_2_-d_4_ (Cayman Chemical, Ann Arbor, MI, USA) as an internal standard was added to 2 ml cell media after which samples were centrifuged (10 min, 4°C, 4,000 × *g*). The supernatant was removed, adjusted to < 10% methanol by the addition of 40 ml water and acidified to pH 3.5 with 1 M HCl. The samples were then applied to a solid phase extraction (SPE) cartridge (C18, 500 mg/6 ml, Biotage, Uppsala, Sweden) conditioned with 2 × 6 ml of methanol and 2 × 6 ml of water. The cartridge was washed with 6 ml water and 2 × 5 ml hexane before elution of the prostaglandin fraction with 2 × 3 ml ethyl acetate. The eluate was evaporated to dryness and reconstituted in 100 µl 50:50 methanol:water (v/v).

The prostaglandins were analysed on a Sciex QTRAP 6500 mass spectrometer (Concord, ON, Canada) interfaced with a Shimadzu Nexera-X2 UHPLC system (Kyoto, Japan). Samples (10 µl) were injected onto Thermo Hypersil Gold C18 column (2.1 mm × 100 mm; 1.9 µm) maintained at 35 °C. Solvent A consisted of 90:10 (v/v) water: methanol with 0.1% acetic acid and solvent B contained methanol with 0.1% acetic acid. The gradient increased from 45–100% solvent B over 18 min and the flow rate was 400 μl/minute. The prostaglandins were identified in negative ion mode using the multiple reaction monitoring (MRM) transitions: PGD_2_ 351 → 271; PGE_2_ 351 → 271; PGF_2α_: 353 → 193; PGJ_2_ 333 → 189; PGE_2_-d_4_ 355 → 275. The concentration of prostaglandins was determined by comparison to a calibration curve run in parallel for each compound and adjusted for recovery by reference to amounts of the internal standard.

### Stable isotope labelling by amino acids in cell culture (SILAC)

For SILAC experiments, cells were cultured as described above with the following modifications. Culture media consisted of DMEM for SILAC (Fisher Scientific, Loughborough, UK*)* supplemented with L-arginine:HCL (^13^C_6_, 99%; ^15^N_4_, 99%) and L-lysine:2HCl (^13^C_6_, 99%) (Cambridge Isotope Laboratories Inc., Tewksbury, MA, USA) and 10% dialysed FBS (Fisher Scientific), 1% penicillin/streptomycin and 1% L-glutamine. Control cells were grown in the labelled media and incubated with PBS for 8 h. Cells treated with Kdo2-lipid A and ATP were grown in the unlabelled media as described above. All cells were collected and pelleted and stored at -20 °C until processed. Cells were lysed and protein extracts from labelled and unlabelled cells mixed in stoichiometric amounts.

### 1-D SDS-PAGE

Proteins from the whole cell lysate (15 μg per condition) were separated by 1-D SDS-PAGE using a Mini Protean Tetra system (Bio-Rad Laboratories Ltd, Hemel Hempstead, UK). Samples were electrophoresed at a constant potential of 200 V using a Bio-Rad PowerPac basic through a 12% (w/v) polyacrylamide resolving gel with a 4% (w/v) stacking gel (Precast TGX Mini-Protean Tetra, Bio-Rad, Walford, UK). Samples were incubated at 90°C for 5 min in a reducing buffer (125 mM Tris hydrochloride (Tris–HCl); 140 mM SDS; 20% (v/v) glycerol; 200 mM dithiolthreitol (DTT) and 30 mM bromophenol blue) prior to loading. Gels were stained with Coomassie Brilliant Blue R250 (Bio-Rad) and de-stained overnight with water.

### In-gel digestion

Gel lanes were cut into 24 slices and each gel piece was incubated at 37°C for 10 min with 100 μl destain solution consisting of 50:50 (v/v) acetonitrile and 100 mM ammonium bicarbonate. This step was repeated twice following which the protein disulfide bonds were reduced by incubating with 50 µl 10 mM DTT at 37˚C for 30 min and the resulting free cysteine residues were alkylated with 50 µl 55 mM iodoacetamide (IAN) at 37˚C for 30 min in the dark. Each gel slice was then dehydrated in acetonitrile (ACN) (100 μL at 37˚C for 15 min) and after evaporating off solvent, proteins were digested at 37˚C using 50 µl 0.1 µg/µl sequencing grade trypsin (Roche) which after 1 h was diluted with 20 µl of 50 mM ammonium bicarbonate and incubated at 37˚C overnight.

### LC–MS/MS analysis of peptides

Peptides were analysed by LC–MS/MS using a Waters nanoAcquity UPLC platform (Milford, MA, USA) coupled to a Thermo LTQ-Orbitrap XL mass spectrometer. The peptides (5 μl) were injected onto a 5 μm, 180 μm × 20 mm Symmetry C18 trapping column (Waters) followed by a 1.7 µm, 75 µm × 250 mm Ethylene Bridged Hybrid (BEH) C18 nanocolumn (Waters). A flow rate of 300 nl/minute was used with an ACN:water gradient with 0.1% formic acid (1% ACN for 1 min, followed by 0–62.5% ACN during 21 min, 62.5–85% ACN for 1.5 min, 85% ACN for 2 min and 100% ACN for 15 min). All analyses were performed in positive ion mode at a resolution of 30,000 over the mass to charge ratio (m/z) range 400–2000 using the lock mass setting. The top 5 precursor ions were automatically isolated and fragmented using collision induced dissociation (CID) energy of 35. Charge state screening was enabled, rejecting ions with unassigned or single positive charge states.

### Proteomic data analysis

Raw files were aligned and processed using Progenesis QI for Proteomics (Nonlinear Dynamics, Newcastle, UK, version 4.0). Data were searched against the SwissPROT Mus muscosa (mouse) database using Mascot (Matrix Sciences, London, UK, version 2.3) to match identified peaks to peptides. The initial search parameters allowed for three trypsin missed cleavages, carbamidomethyl modification of cysteine residues, oxidation of methionine, a precursor mass tolerance of 10 ppm and a fragment mass tolerance of ± 0.8 Da. Additional variable modifications of L-Lysine (^13^C_6_) and L-Arginine (^13^C_6_, ^15^N_4_) were applied for the analysis of stable isotope labelled peptides. Only proteins identified by two or more peptides and a Mascot score larger than 20 were retained. Following peptide identification isotopic data were imported into Proteolabels version 1.1 (Omic Analytics Limited, Leeds, UK), using the detection settings of 10 ppm mass and 0.5 retention time tolerance to detect labelled and unlabelled isotopic peptide pairs. An additional search was undertaken using Proteome Discoverer version 2.4 (Thermo Fisher Scientific) with SEQUEST against the Mus musculus (10,090) FASTA database. The same search parameters were used as with Mascot search with the addition of the dynamic modifications of acetylation, loss of methionine and loss of methionine and acetyl on the protein N-terminus. Normalized labelled and unlabelled abundances were imported into Prism (GraphPad, San Diego, CA, USA, version 6.07). A Wilcoxon-Mann–Whitney test was carried out to identify peptides with significant differences (*p* < 0.05) between their abundances. Peptides were grouped into assigned proteins and the average fold change was determined. Proteins with a minimum of two peptide hits were analysed by gene ontology functional analysis.

### Gene ontology

Gene ontology cluster analysis using ClueGo version 2.33 application within the Cytoscape version 3.5.0 environment was undertaken in order to carry out functional analysis. Proteins which had altered change in relative abundance (< 0.5 or > 2) were analysed with default parameters and an overall statistical significance set to *p* < 0.05 with Bonferroni step-down *p*Value correction. As the quantitative criteria for overexpressed and repressed protein is fold change ratio, the parameters < 0.5 or > 2 are reciprocal (i.e. ½ and 2). To form a more holistic functional analysis, biological, molecular and cellular ontologies were searched along with the KEGG and Reactome databases. 

## Supplementary Information

**Additional file 1:** **Supporting Figure 1.** SILAC protein data and associated gene ontology. The log 2-fold changes in protein abundance between PBS control and [Kdo2-lipid A + ATP] treated RAW264.7 cells are provided (A) and the average fold change is detailed along with any linked ontological classification (B).

**Additional file 2:** **Supporting Figure 2.** KEGG glycolysis/gluconeogenesis pathway. The KEGG pathway for glycolysis/gluconeogenesis is provided/ Proteins with altered expression following treatment are highlighted in orange. KEGG ID: map00010 via KEGGScape [1, 2].

**Additional file 3:** **Supporting Table 1.** Proteins with changes in expression following the induction of inflammation.

**Additional file 4:** **Supporting Table 2.** Eicosanoid and lipid metabolism related proteins. Pathway description provided by Proteome Discoverer 2.4.

## Data Availability

The datasets used and/or analysed during the current study are available from the author NB on reasonable request by emailing nicole.brace@uhi.ac.uk.
