# Supplementary figures and images for: SILAC-based quantitative proteomics to investigate the eicosanoid associated inflammatory response in activated macrophages
Source: J Inflamm (Lond). 2022 Sep 1;19:12. doi: 10.1186/s12950-022-00309-8 (PMC9438320; doi:10.1186/s12950-022-00309-8)

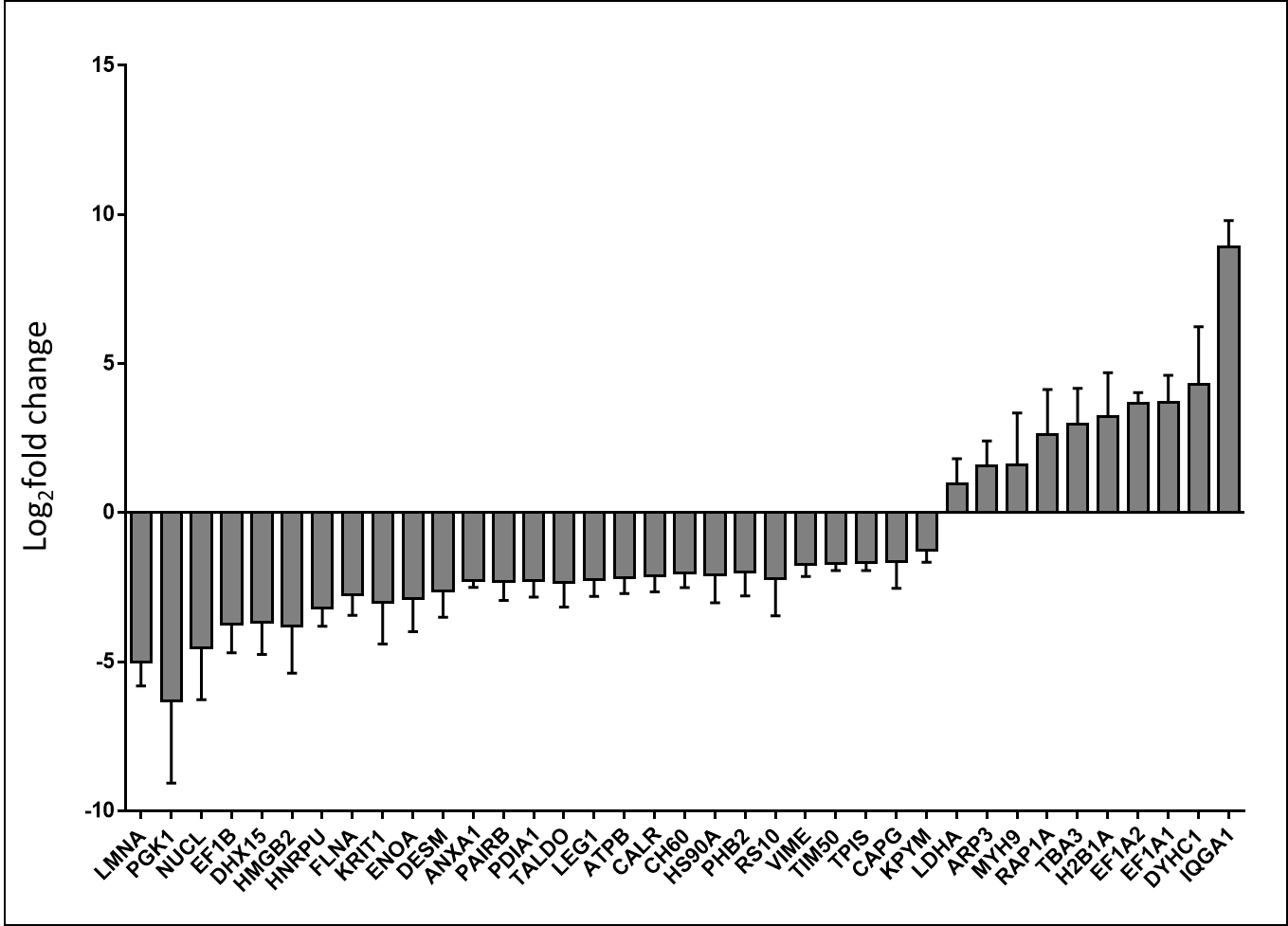

Supplement: Supplementary file 1 — Additional file 1: Supporting Figure 1. SILAC protein data and associated gene ontology. The log 2-fold changes in protein abundance between PBS control and [Kdo2-lipid A + ATP] treated RAW264.7 cells are provided (A) and the average fold change is detailed along with any linked ontological classification (B). [file 12950_2022_309_MOESM1_ESM.zip › Figure S1A.png]

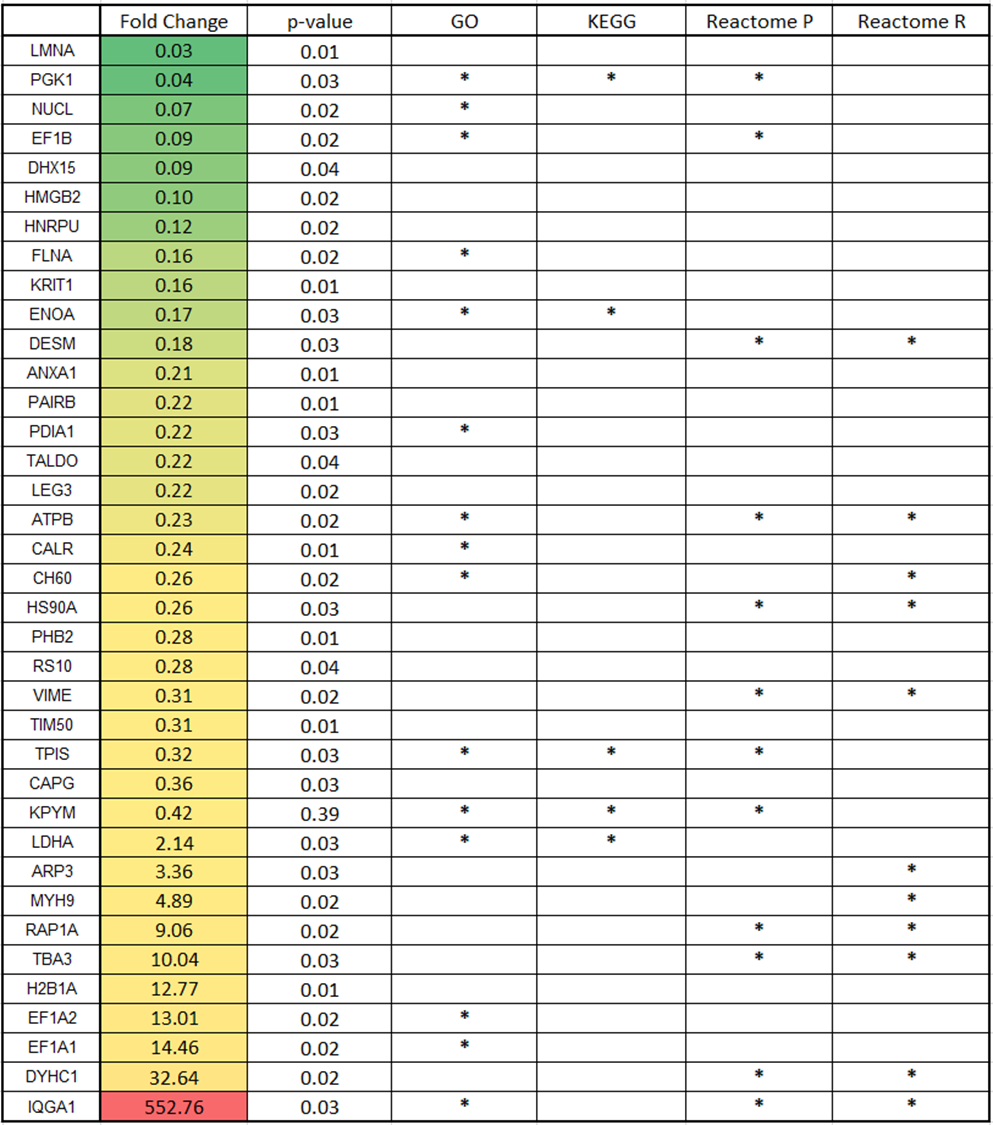

Supplement: Supplementary file 1 — Additional file 1: Supporting Figure 1. SILAC protein data and associated gene ontology. The log 2-fold changes in protein abundance between PBS control and [Kdo2-lipid A + ATP] treated RAW264.7 cells are provided (A) and the average fold change is detailed along with any linked ontological classification (B). [file 12950_2022_309_MOESM1_ESM.zip › Figure S1B.png]

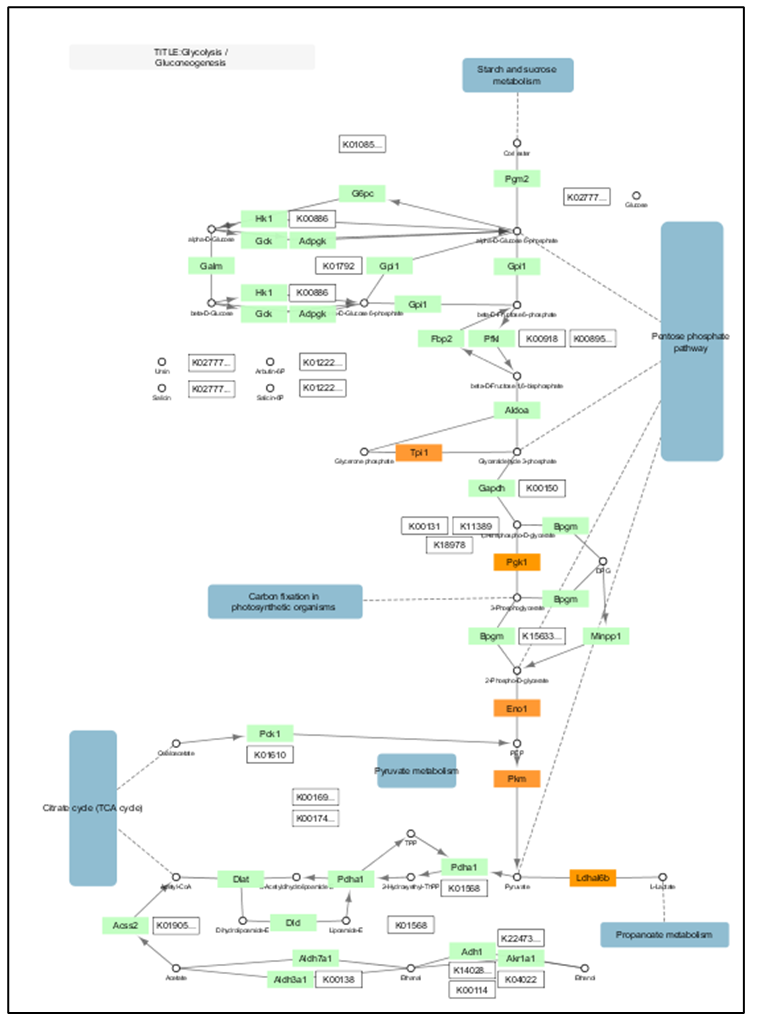

Supplement: Supplementary file 2 — Additional file 2: Supporting Figure 2. KEGG glycolysis/gluconeogenesis pathway. The KEGG pathway for glycolysis/gluconeogenesis is provided/ Proteins with altered expression following treatment are highlighted in orange. KEGG ID: map00010 via KEGGScape [1, 2]. [file 12950_2022_309_MOESM2_ESM.png]
