# Supplementary material for: SILAC-based quantitative proteomics to investigate the eicosanoid associated inflammatory response in activated macrophages
Source: J Inflamm (Lond). 2022 Sep 1;19:12. doi: 10.1186/s12950-022-00309-8 (PMC9438320; doi:10.1186/s12950-022-00309-8)
Supplement: Supplementary file 3 — Additional file 3: Supporting Table 1. Proteins with changes in expression following the induction of inflammation. [file 12950_2022_309_MOESM3_ESM.docx]

| **Uniprot ID** | **Protein name** | **Accession** | **Gene name** | **Score** | **% Coverage** | **Total Peptide Count** |
| --- | --- | --- | --- | --- | --- | --- |
| LMNA | Prelamin-A/C | P48678 | Lmna Lmn1 | 50.73 | 18.35 | 21 |
| PGK1 | Phosphoglycerate kinase 1 | P09411 | Pgk1 Pgk-1 | 38.34 | 27.34 | 32 |
| NUCL | Nucleolin | P09405 | Ncl Nuc | 47.53 | 23.48 | 51 |
| EF1B | Elongation factor 1-beta | O70251 | Eef1b Eef1b2 | 48.18 | 16.92 | 21 |
| DHX15 | Pre-mRNA-splicing factor ATP-dependent RNA helicase DHX15 | O35286 | Dhx15 Ddx15 Deah9 | 47.19 | 6.67 | 6 |
| HMGB2 | High mobility group protein B2 | P30681 | Hmgb2 Hmg2 | 22.57 | 25.24 | 5 |
| HNRPU | Heterogeneous nuclear ribonucleoprotein U | Q8VEK3 | Hnrnpu Hnrpu | 25.48 | 12.00 | 12 |
| FLNA | Filamin-A | Q8BTM8 | Flna Fln Fln1 | 35.72 | 8.95 | 25 |
| KRIT1 | Krev interaction trapped protein 1 | Q6S5J6 | Krit1 Ccm1 | 30.09 | 1.09 | 2 |
| ENOA | Alpha-enolase | P17182 | Eno1 Eno-1 | 52.87 | 35.95 | 31 |
| DESM | Desmin | P31001 | Des | 35.83 | 4.69 | 16 |
| ANXA1 | Annexin A1 | P10107 | Anxa1 Anx1 Lpc-1 Lpc1 | 78.75 | 12.72 | 6 |
| PAIRB | Plasminogen activator inhibitor 1 RNA-binding protein | Q9CY58 | Serbp1 Pairbp1 | 61.11 | 6.63 | 6 |
| PDIA1 | Protein disulfide-isomerase | P09103 | P4hb Pdia1 | 43.37 | 26.72 | 18 |
| TALDO | Transaldolase | Q93092 | Taldo1 Tal Taldo | 45.24 | 34.13 | 14 |
| LEG1 | Galectin-1 | P16045 | Lgals1 Gbp | 32.10 | 17.05 | 11 |
| ATPB | ATP synthase subunit beta, mitochondrial | P56480 | Atp5f1b Atp5b | 56.43 | 51.04 | 46 |
| CALR | Calreticulin | P14211 | Calr | 40.58 | 31.97 | 25 |
| CH60 | 60 kDa heat shock protein, mitochondrial | P63038 | Hspd1 Hsp60 | 56.03 | 44.68 | 52 |
| HS90A | Heat shock protein HSP 90-alpha | P07901 | Hsp90aa1 Hsp86 Hsp86-1 Hspca | 39.83 | 12.96 | 70 |
| PHB2 | Prohibitin-2 | O35129 | Phb2 Bap Bcap37 Rea | 46.79 | 34.78 | 17 |
| RS10 | 40S ribosomal protein S10 | P63325 | Rps10 | 20.59 | 29.70 | 21 |
| VIME | Vimentin | P20152 | Vim | 46.65 | 71.89 | 122 |
| TIM50 | Mitochondrial import inner membrane translocase subunit TIM50 | Q9D880 | Timm50 Tim50 | 47.70 | 7.65 | 4 |
| TPIS | Triosephosphate isomerase | P17751 | Tpi1 Tpi | 55.83 | 46.82 | 35 |
| CAPG | Macrophage-capping protein | P24452 | Capg Mbh1 | 68.16 | 23.01 | 21 |
| KPYM | Pyruvate kinase PKM | P52480 | Pkm Pk3 Pkm2 Pykm | 52.11 | 55.56 | 136 |
| LDHA | L-lactate dehydrogenase A chain | P06151 | Ldha Ldh-1 Ldh1 | 34.50 | 35.84 | 41 |
| ARP3 | Actin-related protein 3 | Q99JY9 | Actr3 Arp3 | 46.10 | 30.86 | 18 |
| MYH9 | Myosin-9 | Q8VDD5 | Myh9 | 49.94 | 27.30 | 92 |
| RAP1A | Ras-related protein Rap-1A | P62835 | Rap1a Krev-1 | 47.48 | 20.11 | 19 |
| TBA3 | Tubulin alpha-3 chain | P05214 | Tuba3a Tuba3; Tuba3b Tuba7 | 58.05 | 28.00 | 77 |
| H2B1A | Histone H2B type 1-A | P70696 | H2bc1 Hist1h2ba Th2b | 37.25 | 54.33 | 141 |
| EF1A2 | Elongation factor 1-alpha 2 | P62631 | Eef1a2 Eef1al Stn | 39.99 | 56.71 | 79 |
| EF1A1 | Elongation factor 1-alpha 1 | P10126 | Eef1a1 | 31.99 | 25.05 | 63 |
| DYHC1 | Cytoplasmic dynein 1 heavy chain 1 | Q9JHU4 | Dync1h1 Dhc1 Dnch1 Dnchc1 Dyhc | 46.36 | 4.26 | 17 |
| IQGA1 | Ras GTPase-activating-like protein IQGAP1 | Q9JKF1 | Iqgap1 | 55.42 | 7.36 | 24 |
