# Supplementary material for: SILAC-based quantitative proteomics to investigate the eicosanoid associated inflammatory response in activated macrophages
Source: J Inflamm (Lond). 2022 Sep 1;19:12. doi: 10.1186/s12950-022-00309-8 (PMC9438320; doi:10.1186/s12950-022-00309-8)
Supplement: Supplementary file 4 — Additional file 4: Supporting Table 2. Eicosanoid and lipid metabolism related proteins. Pathway description provided by Proteome Discoverer 2.4. [file 12950_2022_309_MOESM4_ESM.docx]

| Accession | Uniprot | Protein Name | Fold Change  (observed in ‘n’ samples) | Pathway Description |
| --- | --- | --- | --- | --- |
| P12787 | COX5A | Cytochrome c oxidase subunit 5A | 100  (n=3) | Arachidonate Epoxygenase,  Epoxide Hydrolase |
| P48036 | ANXA5 | Annexin A5 | 21  (n=3) | Prostaglandin Synthesis and Regulation |
| P14069 | S10A6 | Protein S100-A6 | 1.6  (n=1) | Prostaglandin Synthesis and Regulation |
| O35639 | ANXA3 | Annexin A3 | 2  (n=2) | Prostaglandin Synthesis and Regulation,  Phospholipase inhibitor activity, Phospholipase inhibitor activity |
| Q8BMS1 | ECHA | Trifunctional enzyme subunit alpha, | 3.2  (n=1) | Phospholipid metabolism,  Metabolism of Lipids |
| P84104 | SRSF3 | Serine/arginine-rich splicing factor 3 | 3.9  (n=1) | Phospholipase binding,  MyD88 cascade initiated on plasma membrane |
| P14733 | LMNB1 | Lamin-B1 | 72  (n=3) | Phospholipase binding |
| Q91YR9 | PTGR1 | Prostaglandin reductase 1 | 100  (n=1) | 15-oxoprostaglandin and 13-oxidase activity,  Prostanoid metabolic process, Prostaglandin metabolic process |
| P63017 | HSP7C | Heat shock cognate 71 kDa protein | 2  (n=2) | Prostaglandin binding |
| P63038 | CH60 | 60 kDa heat shock protein, mitochondrial | 34  (n=3) | Lipopolysaccharide receptor complex |
| P63158 | HMGB1 | High mobility group protein B1 | 100  (n=1) | Regulation of toll-like receptor 4 signaling pathway |
| P19096 | FAS | Fatty acid synthase | 4.2  (n=3) | Metabolism of lipids |
| P45377 | ALD2 | Aldose reductase-related protein 2 | 25  (n=3) | Metabolism of lipids |
| Q61820 | RANT | GTP-binding nuclear protein Ran | 4  (n=1) | Metabolism of lipids |
| Q8BYH8 | CHD9 | Chromodomain-helicase-DNA-binding protein 9 | 51  (n=2) | Metabolism of lipids |
| E9QNN1 | E9QNN1 | ATP-dependent RNA helicase A | 35  (n=3) | MyD88 cascade initiated on plasma membrane,  Toll like receptor 4 (TLR4) cascade |
